# MpeV is a lyase isomerase that ligates a doubly linked phycourobilin on the β-subunit of phycoerythrin I and II in marine *Synechococcus*

**DOI:** 10.1074/jbc.RA120.015289

**Published:** 2020-11-23

**Authors:** Lyndsay A. Carrigee, Jacob P. Frick, Jonathan A. Karty, Laurence Garczarek, Frédéric Partensky, Wendy M. Schluchter

**Affiliations:** 1 Department of Biological Sciences, University of New Orleans, New Orleans, Louisiana, USA; 2 Department of Chemistry, Indiana University, Bloomington, Indiana, USA; 3 Ecology of Marine Plankton (ECOMAP) Team, Station Biologique, Sorbonne Université & CNRS, UMR 7144, Roscoff, France

**Keywords:** bilin lyase, cyanobacteria, lyase isomerase, phycoerythrobilin, phycobilisome, phycourobilin, posttranslational modification, C, cysteine residue, CA4, Type IV chromatic acclimation, CpeA/CpeB, α-/β-subunit of phycoerythrin type I, EICs, extracted ion chromatograms, HT-, hexahistidine-tagged, ML, Maximum Likelihood, MpeA/MpeB, α-/β-subunit of phycoerythrin type II, MS, mass spectrometry, MW, molecular weight, PAGE, polyacrylamide gel electrophoresis, PBP, phycobiliprotein(s), PBS, phycobilisome(s), PEI, phycoerythrin I, PEII, phycoerythrin II, PEB, phycoerythrobilin, PDB, Protein Data Bank, PUB, phycourobilin, SDS, sodium dodecyl sulfate

## Abstract

*Synechococcus* cyanobacteria are widespread in the marine environment, as the extensive pigment diversity within their light-harvesting phycobilisomes enables them to utilize various wavelengths of light for photosynthesis. The phycobilisomes of *Synechococcus* sp. RS9916 contain two forms of the protein phycoerythrin (PEI and PEII), each binding two chromophores, green-light absorbing phycoerythrobilin and blue-light absorbing phycourobilin. These chromophores are ligated to specific cysteines *via* bilin lyases, and some of these enzymes, called lyase isomerases, attach phycoerythrobilin and simultaneously isomerize it to phycourobilin. MpeV is a putative lyase isomerase whose role in PEI and PEII biosynthesis is not clear. We examined MpeV in RS9916 using recombinant protein expression, absorbance spectroscopy, and tandem mass spectrometry. Our results show that MpeV is the lyase isomerase that covalently attaches a doubly linked phycourobilin to two cysteine residues (C50, C61) on the β-subunit of both PEI (CpeB) and PEII (MpeB). MpeV activity requires that CpeB or MpeB is first chromophorylated by the lyase CpeS (which adds phycoerythrobilin to C82). Its activity is further enhanced by CpeZ (a homolog of a chaperone-like protein first characterized in *Fremyella diplosiphon*). MpeV showed no detectable activity on the α-subunits of PEI or PEII. The mechanism by which MpeV links the A and D rings of phycourobilin to C50 and C61 of CpeB was also explored using site-directed mutants, revealing that linkage at the A ring to C50 is a critical step in chromophore attachment, isomerization, and stability. These data provide novel insights into β-PE biosynthesis and advance our understanding of the mechanisms guiding lyase isomerases.

Marine cyanobacteria in the genus *Synechococcus* are the second most abundant oxygenic phototrophs and contribute significantly to global ocean primary productivity and carbon cycling (1). *Synechococcus* are widespread in part because of their efficiency at harvesting available light using their antenna or phycobilisome (PBS), which is tuned to absorb light colors from portions of the visible spectrum in which chlorophyll absorbs poorly (2, 3, 4). Marine isolates of *Synechococcus* have a complex PBS structure comprised of up to four highly pigmented phycobiliproteins (PBP). The PBS core is made of allophycocyanin and is surrounded by six to eight rods made of phycocyanin and up to two types of phycoerythrin (PEI and PEII). These extended rod structures increase the spectral range of the PBS light harvesting capabilities (Fig. 1) (1, 3, 5, 6, 7). PEI and PEII are homologous PBP, each composed of an α- and a β-subunit arranged in a hetero-hexameric (αβ)_6_ torus and stacked with the help of linker polypeptides to form the distal portion of the rods (Fig. 1) (4, 8, 9). The large pigment diversity of the PBS is not only due to its variable PBP content but also to the variable proportion of covalently bound linear tetrapyrrole bilins, posttranslationally added to PBP. Highly conserved cysteine (C) residues of PEI and PEII serve as the sites for covalent attachment of bilins *via* the activity of specialized enzymes known as bilin lyases.

**Figure 1:**
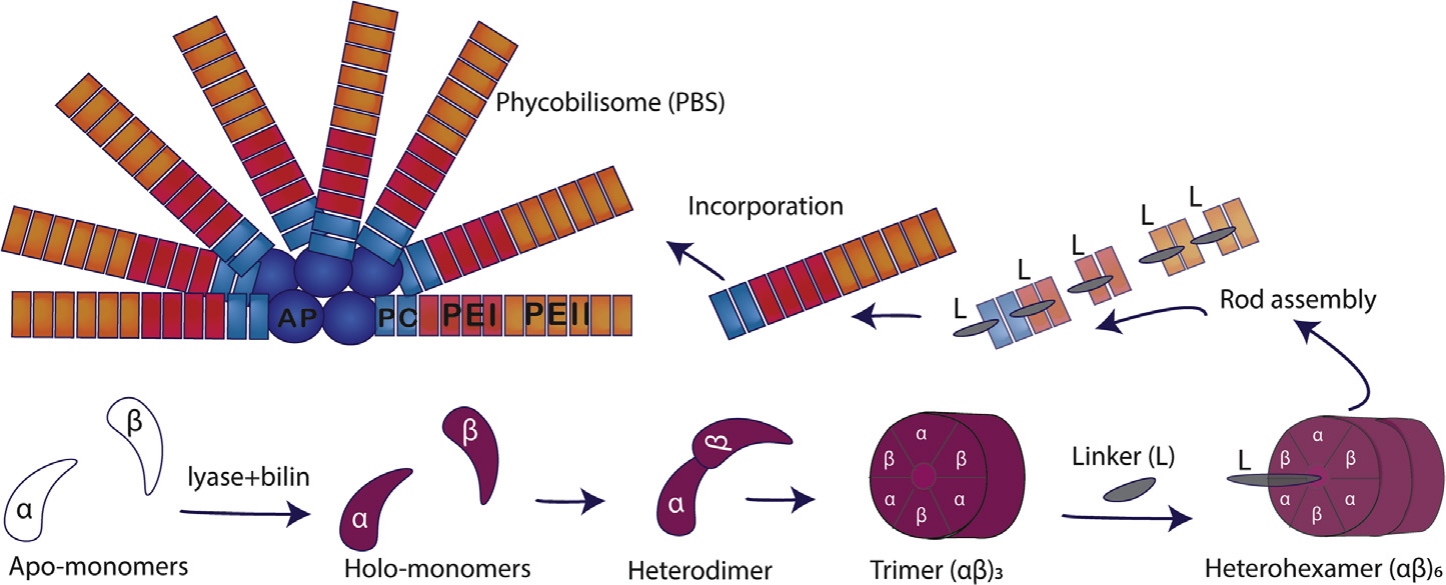
**Model of *Synechococcus* sp. RS9916 PBS rod assembly.** Model of an RS9916 phycobilisome (PBS) containing phycoerythrin I (PEI, *red*), phycoerythrin II (PEII, *orange*), phycocyanin (PC, *light blue*), and an allophycocyanin (AP, *deep blue*) core. *A depiction of PBS rod* assembly shows the addition of bilin *via* posttranslational modification of apo-α and apo-β monomers (*white*) forming holo-monomers (*purple*), which come together to form a heterodimer. Heterodimers are subsequently arranged in trimers (αβ)_3_ followed by heterohexamers (αβ)_6_ and with the help of linker polypeptides, the PBS rod is formed and bound onto the core (4, 49, 65, 66).

Based on sequence similarities, three major groups or clans of bilin lyases have been characterized: CpcS/U type, CpcT type, and CpcE/F type, (10, 11, 12, 13, 14). Each clan differs from one another in primary amino acid sequence and structure as well as bilin chromophore and attachment site specificity. Solved crystal structures for members of the distantly related CpcS/U (Protein Data Bank, (PDB): 3BDR; (4, 15, 16, 17, 18)) and CpcT (PDB: 4O4O; (19, 20)) lyase families show that they adopt a similar antiparallel beta-barrel structure. CpcS-type lyases are hypothesized to have evolved first because they recognize the central C82-equivalent position present in α and β subunits of allophycocyanin, β-phycocyanin, and β-PE (4, 10, 11, 15, 19, 20, 21, 22). Unrelated to the other clans, the CpcE/F lyase clan members display a high specificity for a single bilin and a single binding site on a particular PBP (PDB 5N3U; (13, 14, 23, 24)). These lyases contain five to six HEAT-repeat motifs (thought to facilitate protein–protein interactions) coupled with Armadillo repeats (25, 26, 27, 28) (Fig. S1) (4, 22, 29). This CpcE/F group also includes enzymes that have both bilin isomerase and ligase activity (lyase isomerases), proteins with chaperone-like functions, and proteins with the capability to remove bilins (13, 14, 30, 31, 32, 33, 34). To date, the crystal structure of only one CpcE/F-type lyase has been solved and was found to adopt an alpha helical solenoid shape (29).

The marine *Synechococcus* sp. strain RS9916 possesses the ability to alter the ratio of the blue-light-absorbing chromophore phycourobilin (PUB) [absorbance maximum (λ_max_) ∼495 nm] and the green-light-absorbing chromophore phycoerythrobilin (PEB) (λ_max_ ∼545 nm) on the distal portion of the PBS rods, a phenomenon known as type IV chromatic acclimation (CA4) (3, 6, 35, 36, 37, 38, 39). During CA4, the PUB-to-PEB ratio (PUB:PEB) is adjusted to be higher in blue light and lower in green light in order to optimize light capture in changing light color environments (3, 31, 35, 36, 39, 40). All CA4 strains contain one of the two genomic island configurations, CA4-A or CA4-B, each encoding transcritional activators and a lyase or a lyase isomerase (36). Although PEB is readily produced in cyanobacteria as a free precursor molecule (41), the bilin PUB is produced through the activity of specialized bilin lyase isomerases that attach PEB while simultaneously isomerizing it to PUB (Fig. 2) (31, 42). Increased PUB content in PBS allows these organisms to better absorb the blue light available in deeper water (43).

**Figure 2:**
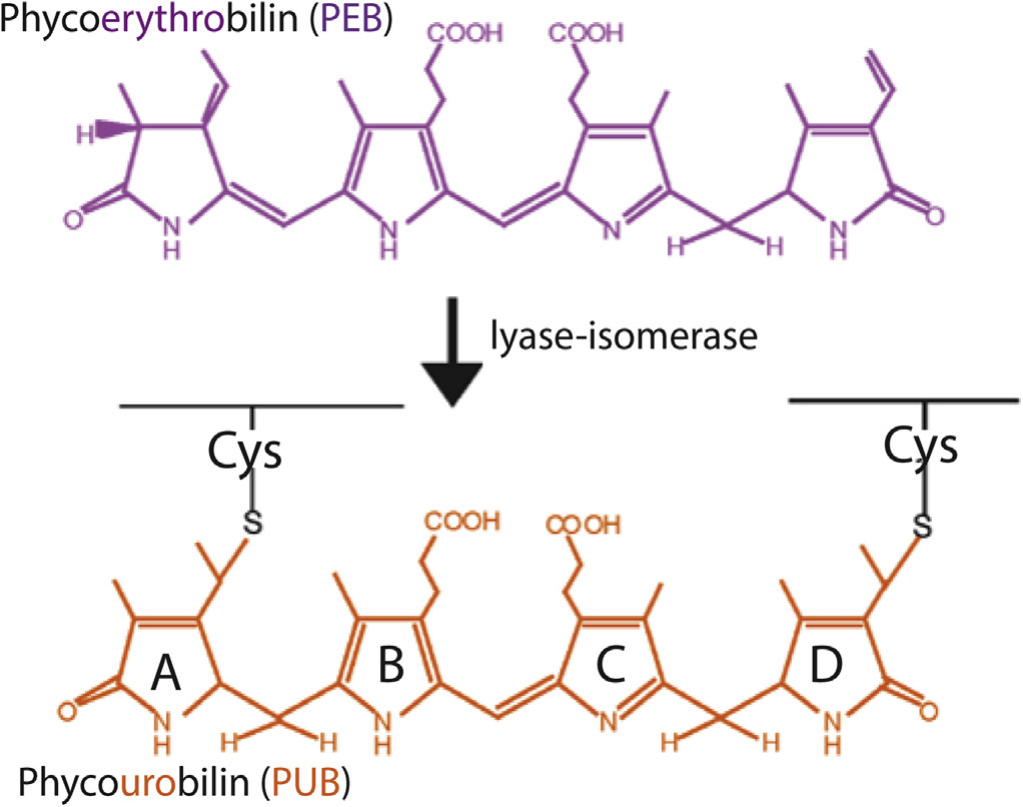
**Chemical structures of phycoerythrobilin (PEB) and doubly linked phycourobilin (PUB).** Posttranslational pigment attachment is catalyzed by bilin lyases or lyase isomerases with a thioether linkage at the 3^1^ carbon of the bilin A ring during single attachment or additionally through the 18^1^ carbon of the bilin D ring when doubly attached.

In RS9916, there are five possible sites for bilin attachment on PEI, two on the α-subunit (CpeA) and three on the β-subunit (CpeB), and six possible sites on PEII, three each on the α-subunit (MpeA) and β-subunit (MpeB) (31). It is hypothesized that a bilin lyase is responsible for ligation of a given bilin at each individual site. Thus far, only four PE lyases (all members of the CpcE/CpcF clan) have been characterized for RS9916 in the literature (31, 38, 43, 44). During CA4, MpeY (PEB lyase) and MpeZ (PUB lyase isomerase) chromophorylate the C83 position of MpeA in green or blue light, respectively (31, 38). CpeY adds PEB to the C82 position of CpeA, a site of constitutive attachment, not involved in any of the major CA4 changes that occur (44). MpeU is responsible for PUB attachment in BL (43, 45); however, more research is needed to fully understand its function and potential role in chromatic acclimation. To date, no lyases for β-PEI or β-PEII have been characterized in RS9916, but some studies on the biosynthesis of CpeB from the freshwater cyanobacterium *Fremyella diplosiphon* have been completed (46). In *F. diplosiphon*, CpeS is the PEB lyase for C82 on CpeB and CpeF is the PEB lyase for the doubly ligated PEB at C50,61, and both of these enzymes require the chaperone-like protein CpeZ to keep the CpeB substrate soluble (34, 46). In these recent studies, Kronfel *et al.* (46) also characterized the chromophorylation pattern for the doubly linked PEB at C50, 61 in *F. diplosiphon* CpeB by CpeF and demonstrated that linkage along the A ring likely occurs first and is important for subsequent attachment to the D ring. The closest homolog to the *cpeF* gene from *F. diplosiphon* in RS9916 is *mpeV*, first described in the genome of marine *Synechococcus* strain WH8020 (47), though WH8020 MpeV has not yet been characterized. However, the presence of a PUB chromophore at the C50, 61 position of the β-subunits of both PEI and PEII in RS9916 (31) led us to question the function of this gene. Here, we demonstrate that RS9916 MpeV is the lyase isomerase responsible for the doubly linked PUB on RS9916 β-PEI (CpeB) and β-PEII (MpeB), and its activity requires ligation of PEB at C82 by CpeS and is enhanced by CpeZ. We also show that linkage of C50 to the A-ring is required for bilin isomerization to occur.

## Results

### Structural prediction

**Figure 3:**
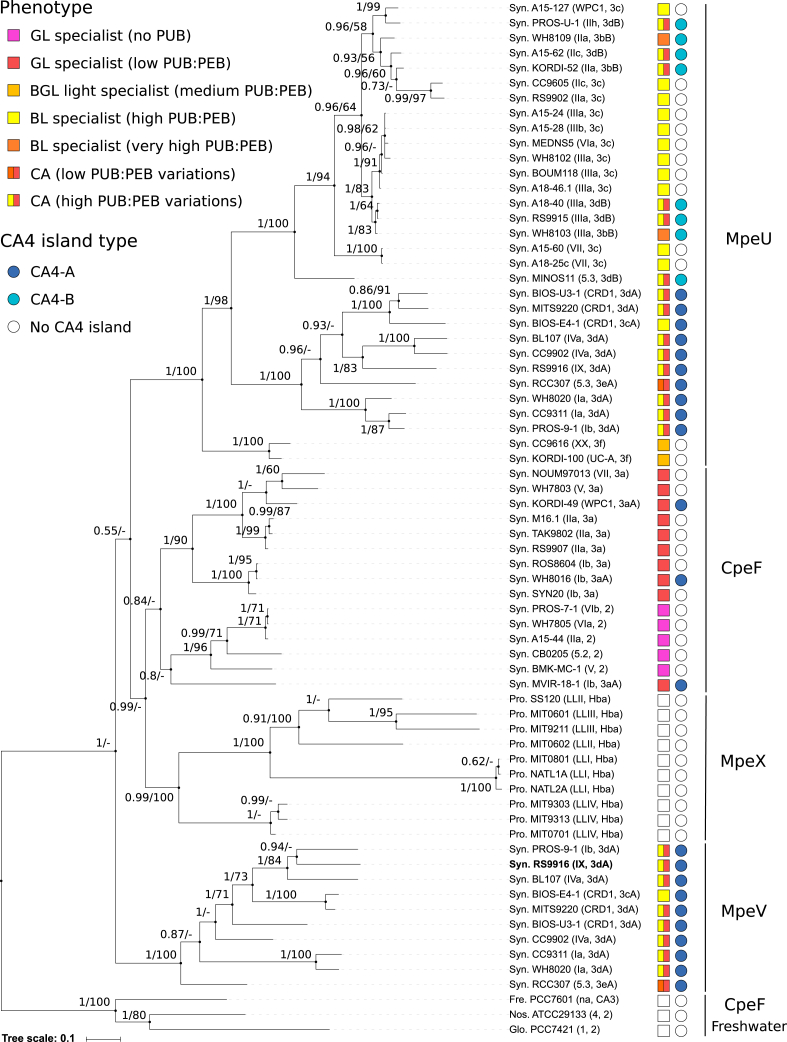
**Phylogenetic tree of the MpeV enzyme family.** Sequence names include abbreviation of the genus (see below), the strain name, the finest taxonomical level for each strain *sensu* (67), *i.e.*, subcluster (*e.g.*, 5.2), clade (*e.g.*, VII), or subclade (*e.g.*, IIIa), as well as the pigment type *sensu* (40). The pigment phenotype of each strain is indicated by a colored square and the CA4-island type (A or B) by a blue circle. Strains called “BGL specialists” (*light orange square*) correspond to strains that are genetically similar to pigment type 3 dB (a.k.a. CA4-B) but that have lost the ability to perform CA4 (*i.e.*, natural CA4 mutants) and are stuck in some intermediate state between the *green* and *blue* light phenotype (35). Also worth noting, several strains with a low PUB:PEB ratio have acquired a complete or partial CA4-A island by lateral transfer (pigment type 3aA), but none is CA4-capable (35). Series of two numbers at nodes of the tree correspond to Bayesian posterior probabilities (PP, ranging between 0 and 1) and bootstrap values for Maximum Likelihood (ML), respectively. Only values higher than 50% for ML bootstrap values and 0.50 for PP are shown on the Bayesian tree. The *Synechococcus* sp. RS9916 strain used in the present study is indicated in bold. Abbreviations for genus names: Fre., *Fremyella*; Glo., *Gloeobacter*; Nos., *Nostoc*; Pro., *Prochlorococcus*; Syn., *Synechococcus*. Other abbreviations: BL, Blue light; BGL, Blue-green light; CA, Chromatic acclimaters; CA4, Chromatic acclimation type IV; GL, Green light; PEB, Phycoerythrobilin; PUB, Phycourobilin.

The putative lyase MpeV of the marine *Synechococcus* strain RS9916 shares sequence similarity (54.9%) with the recently characterized CpeF lyase from the freshwater cyanobacterium *F. diplosiphon*, and a structural prediction analysis using Phyre^2^ shows that they have similar predicted structures (Fig. S1; closest characterized structure PDB: bgfp-a) (48), suggesting that these enzymes may have a similar substrate specificity for β-PE subunits. Yet, RS9916 has PUB doubly linked at C50, 61 of CpeB and MpeB (31), rather than PEB at the equivalent C48, 59 positions of *F. diplosiphon* CpeB (46). This led us to hypothesize that MpeV could be a lyase isomerase acting at C50, 61 on CpeB and perhaps also on MpeB. Of note, in some marine strains of *Synechococcus*, the β-subunits contain PEB in place of PUB at C50, 61 on CpeB, similar to *F. diplosiphon* (7). These strains are either lacking PE-II (pigment type 2 *sensu* (49)) or possess PE-II but exhibit a low PUB:PEB ratio (pigment type 3a), *i.e.*, the so-called “green light specialists.” Therefore, for these strains, we will call these lyases CpeF hereafter (see also Fig. 3), to distinguish them from MpeV of RS9916.

### Comparative genomics and phylogenetic analyses

MpeV was first suggested as a putative lyase by Wilbanks and Glazer, after sequencing a large fraction of the PBS rod genomic region from *Synechococcus* sp. WH8020 (47) (Fig. S2). This MpeV enzyme is specific to marine *Synechococcus* that, like RS9916 and WH8020, are typical CA4-A strains, *i.e.*, possess a CA4-A genomic island and are capable of CA4 (Fig. 3). A notable exception is BIOS-E4-1, a strain that lacks the CA4 regulators FciA and FciB and is a natural CA4-incapable mutant (35, 40). Besides the CpeF lyase found both in phycoerythrin-containing freshwater cyanobacteria, like *F. diplosiphon* (46), and its functional homologs in “green light specialists” (see above), the MpeV family also includes MpeU, which was recently partially characterized as a PE-II-specific lyase isomerase in RS9916 (43, 45). Like *mpeV*, the *mpeU* gene was first reported from the PBS rod genomic region of WH8020 (47). MpeU is found in all strains exhibiting a high PUB:PEB ratio (pigment type 3c), so-called blue light specialists, as well as in strains having a variable PUB:PEB ratio (CA4-A and CA4-B strains; Fig. 3). The last member of this enzyme family is the yet-uncharacterized putative lyase MpeX, found only in low light-adapted *Prochlorococcus marinus* strains (50).

A phylogenetic tree made using representative amino acid sequences of these different lyases and rooted using CpeF sequences from freshwater cyanobacteria (including *F. diplosiphon*) clearly shows that all the subfamilies make distinct branches in the phylogenetic tree (Fig. 3), indicating that they have long diverged from each other, a divergence potentially associated with functional changes. We hypothesize that members of this enzyme family present in marine *Synechococcus* or *Prochlorococcus* strains have all derived from a CpeF-like ancestor, potentially coming from a freshwater cyanobacterium. Although MpeV is found at the base of all marine picocyanobacterial lyases in the tree, its position as well as that of other deep branches has a fairly low bootstrap support, and it is difficult to say with certainty in which order the different subfamilies have occurred during evolution, and notably when the duplication event happened that led to the paralogous *mpeV* and *mpeU* genes, co-occurring in all present-day CA4-A strains (Fig. 3).

### Analysis of recombinant *Synechococcus* sp. RS9916 MpeV, CpeS, and CpeZ on CpeB and MpeB

We sought to determine the function of MpeV using our heterologous *Escherichia coli* expression system. RS9916 genes of interest were expressed using compatible vectors as outlined in Table S1. All coexpressions analyzing β-subunits as substrate were designed to also express α-subunits in an effort to increase solubility of β-subunits (46, 51). Previous work with *F. diplosiphon* showed that CpeS and CpeZ were required to obtain enough chromophorylated, soluble CpeB substrate to allow for measurable CpeF activity (46). Therefore, these genes from RS9916 were included in trials for MpeV activity. Though α-subunits (CpeA/MpeA) copurified with their respective β-subunits (CpeB/MpeB), neither CpeA nor MpeA was chromophorylated by the available lyases (see also LC-MS-MS results section below).

**Figure 4:**
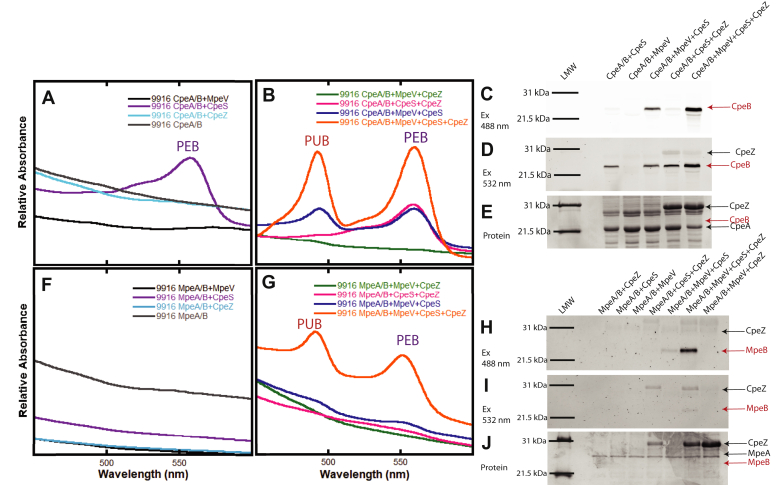
**Recombinant protein activity of RS9916 MpeV on CpeB and MpeB.** Relative absorbance representing purified recombinant RS9916 CpeB (*A*–*B*) and MpeB (*F*–*G*) expressed in the presence/absence of putative lyases MpeV, CpeS, and/or CpeZ, as indicated in the legend inset. All proteins were induced in *E. coli* cells with bilin synthesis genes, purified and diluted to similar concentrations prior to analysis. Purified CpeB (*C*–*E*) and MpeB (*H*–*J*) were resolved *via* SDS-PAGE and imaged with zinc-enhanced fluorescence at 460 to 490 nm (*C* and *H*), which excites PUB and at 520 to 545 nm (*D* and *I*), which excites PEB. The same gels were then stained with Coomassie blue (*E* and *J*) to visualize proteins. Positions of target substrates CpeB and MpeB in gels are indicated by *red arrows*. This data is representative of three independent biological replicates.

Recombinant CpeB purified from coexpressions without a lyase was similar to samples containing MpeV alone, CpeZ alone, or MpeV with CpeZ, showing no detectable bilin addition (Fig. 4*A*) and little soluble CpeB produced (data not shown). Thus these samples were excluded from further analyses. The lyase CpeS is able to attach PEB to CpeB (Fig. 4*A*) as indicated by an absorbance peak at 559 nm, and its activity on CpeB increases in efficiency when coexpressed with CpeZ, a homolog of the characterized chaperone-like protein from *F. diplosiphon* (34) (see Fig. 4*B*). This can be compared directly after zinc-enhanced bilin fluorescence showing bound PEB (Fig. 4*C*). Once CpeB is chromophorylated by CpeS, the β-subunit is soluble enough that MpeV can act. MpeV is able to isomerize PEB to PUB and ligate it to CpeB as indicated by the peak at 493 nm (Fig. 4*B*, blue line), and the efficiency of both CpeS and MpeV activity is increased when coexpressed with CpeZ, as indicated by increased absorbance (Fig. 4*B*, orange line) and zinc-enhanced bilin fluorescence of PUB and PEB (Fig. 4, *C*–*D*).

Next, we wanted to test these enzymes on the MpeB subunit (Fig. 4, *F*–*J*). MpeB is much less soluble than CpeB in our recombinant system. Very little MpeB is chromophorylated as judged by zinc-enhanced bilin fluorescence and absorbance until MpeV and CpeS are added (Fig. 4, *F*–*J*). However, when CpeZ was added with both MpeV and CpeS, a substantial improvement in chromophorylation is achieved, as indicated by the presence of PUB at 493 nm and PEB at 559 nm (Fig. 4*G*). This is corroborated by evidence of both bilins being present from zinc-enhanced fluorescence (Fig. 4, *H*–*I*). Therefore, MpeV is a lyase isomerase, and CpeS is a lyase acting on MpeB. This is the first demonstration of any lyase activity on β-phycoerythrin II subunits.

### LC-MS-MS analysis of recombinant RS9916 proteins

**Figure 5:**
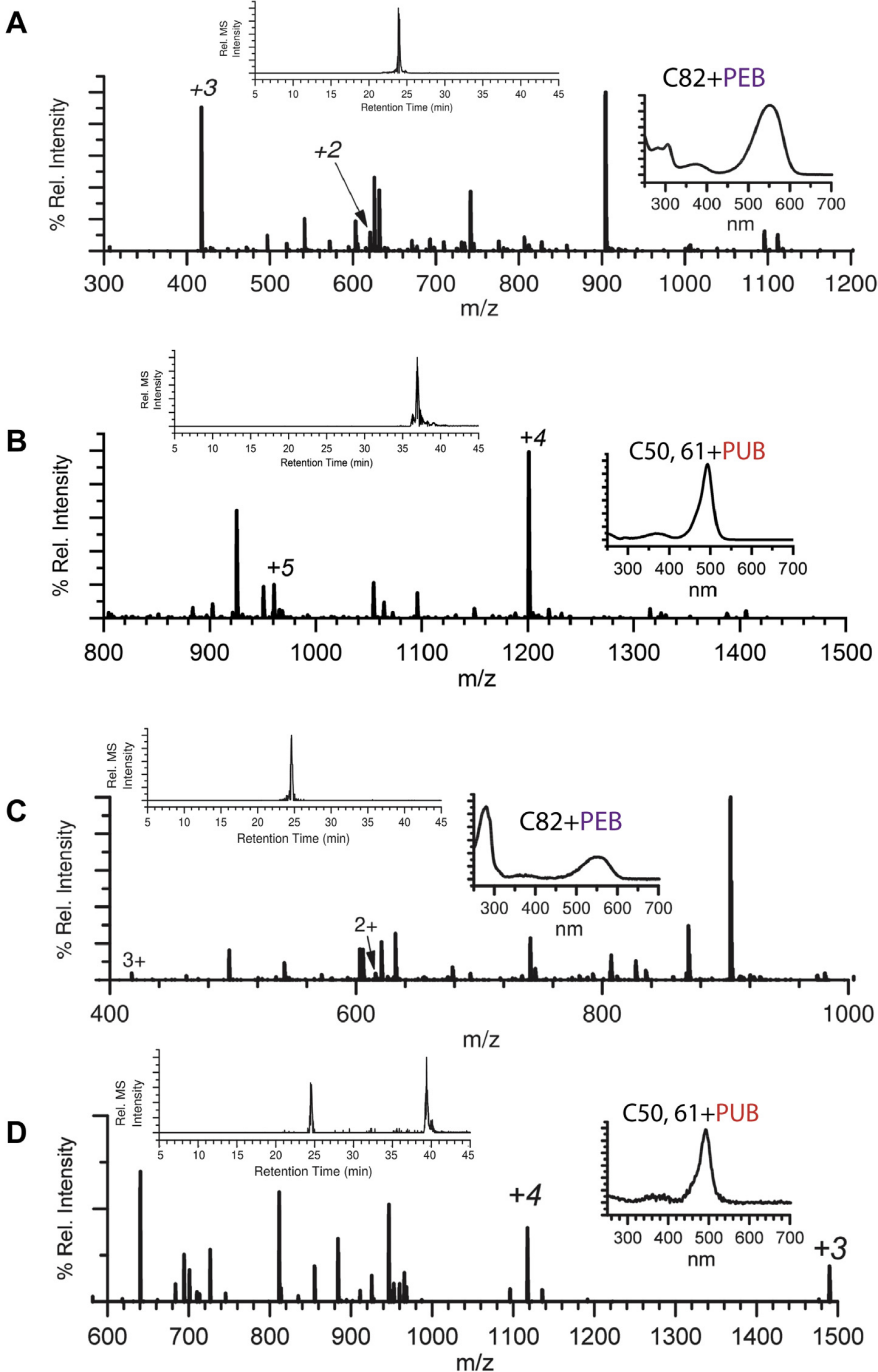
**Extracted ion chromatograms and LC-MS spectra for trypsin-digested peptides from RS9916 recombinant β-subunits.***A*, extracted ion chromatogram (EIC, inset left) and LC-MS for the peptide MAAC_82_LR at *m/z* 417.5^3+^ and 625.8^2+^ of recombinant RS9916 CpeB C82-PEB (∼560 nm). *B*, EIC (inset left) and LC-MS for peptide LDAVNAITSNASC_50_IVSDAVTGMIC_61_ENTGLIQAGGNCYPNRR at *m/z* 1200.2^4+^ and 960.46^5+^ of recombinant RS9916 CpeB C50, 61-PUB (∼490 nm). *C*, EIC (inset left) and LC-MS for the peptide KMAAC_82_LRU at *m/z* 417.5^3+^ and 689.5^2+^ of recombinant RS9916 MpeB C82-PEB (∼560 nm). *D*, EIC (inset left) and LC-MS for peptide LDAVNAIAGNAAC_50_IVSDAVAGICC_61_ENTGLTAPNGGVYTNR at *m/z* 1116.8^4+^ and 1489.7^3+^ of recombinant RS9916 MpeB C50, 61-PUB (∼490 nm). Inset right graphs are the UV–visible absorbance spectra for the peaks present in the EIC (inset left). The type of bilin is indicated per panel. All samples expressed in the presence of CpeS, MpeV, CpeZ, and bilin synthesis genes. These results are representative of two independent biological replicates.

Recombinant CpeB and MpeB proteins expressed in the presence of CpeS, MpeV, and/or CpeZ were purified, digested with trypsin, and subjected to LC-MS-MS. Modification of CpeB-C82 and MpeB-C82 by addition of PEB at these sites (by the lyase CpeS) is detected (Fig. 5, *A* and *C*, Tables 2 and 3; S2 and S3). The UV-VIS spectrum (Fig. 5*A*) clearly demonstrates that PEB is attached to CpeB (∼560 nm absorbance trace). Figure 5*A* shows the extracted ion chromatogram (EIC; inset left) and LC-MS for the peptide MAAC_82_∗LR at *m/z* 417.5^3+^ and 625.8^2+^ of recombinant RS9916 CpeB C82-PEB.

Modification of CpeB-C50, 61 and MpeB-C50, 61 with a doubly ligated PUB by MpeV is also detected (Fig. 5, *B* and *D*, Tables 2 and S2). Figure 5*B* shows the EIC (inset left) and LC-MS for the peptide LDAVNAITSNASC_50_∗IVSDAVTGMIC_61_∗ENTGLIQAGGNCYPNRR at *m/z* 1200.2^+4^ and 960.46^+5^ of recombinant RS9916 CpeB with a doubly linked PUB at C50, 61. The UV-VIS spectrum also demonstrates that PUB is attached to the peptide (∼493 nm absorbance trace). Ions from bilin modified peptides containing C165 from CpeB, C139 from CpeA, or C82 from CpeA were not observed by LC-MS-MS.

Figure 5*C* shows the EIC (inset left) and LC-MS for the peptide MAAC_82_∗LR at *m/z* 417.5^3+^ and 625.8^2+^ of recombinant RS9916 MpeB with PEB at C82 also detectable in the absorbance trace with a peak ∼560 nm. Figure 5*D* shows the EIC (inset left) and LC-MS for peptide LDAVNAIAGNAAC_50_∗IVSDAVAGICC_61_∗ENTGLTAPNGGVYTNR at *m/z* 1117.5^4+^ and 1489.7^+3^ of recombinant RS9916 MpeB C50, 61-PUB (∼490 nm absorbance trace). Ions from bilin modified peptides containing C159 from MpeB or C75, C83, or C140 from MpeA were not observed by LC-MS-MS.

### Analyses of MpeV mechanism of PUB attachment using site-directed mutant variants

**Figure 6:**
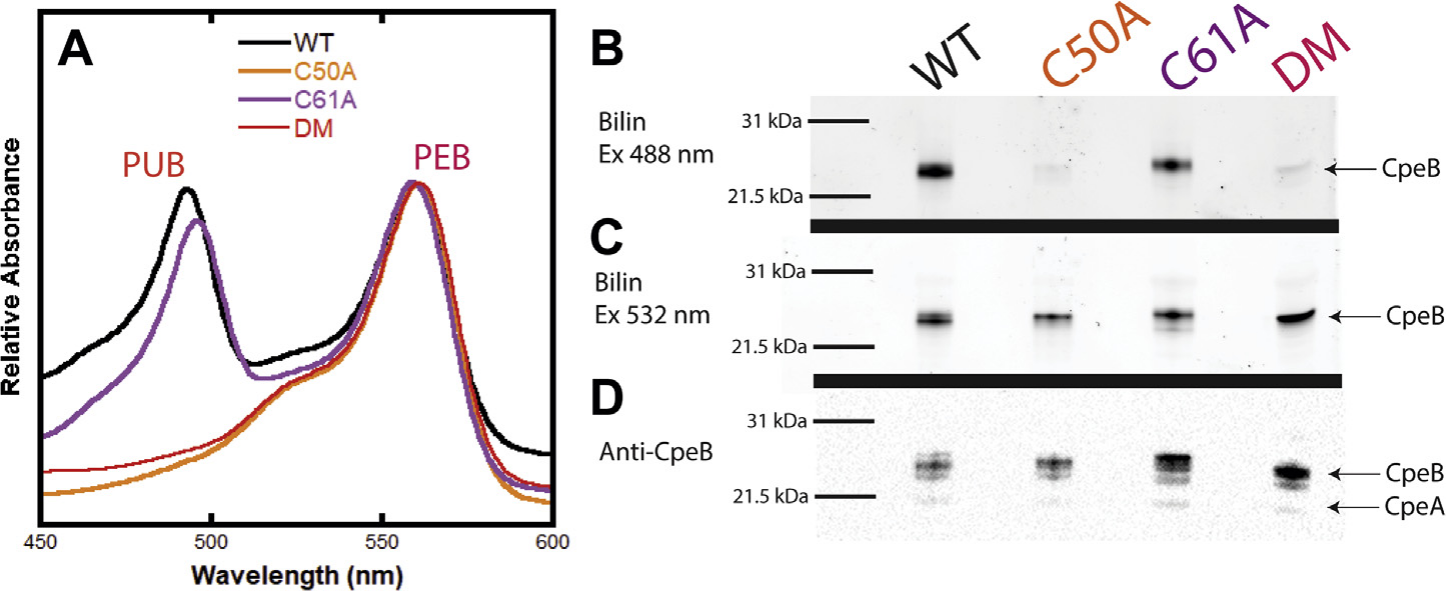
**Recombinant protein activity of MpeV on RS9916 CpeB mutant variants.** Recombinant protein coexpression of RS9916 CpeB and mutant variants in the presence of CpeA, MpeV, CpeS, and CpeZ for maximum solubility and chromophorylation. All purified protein samples were expressed in the presence of bilin synthesis genes. *A*, relative absorbance of nonmutated CpeB, denoted as wild-type (WT; *black line*), shows addition of phycoerythrobilin (PEB) to C82 with an absorbance peak at 559 nm and addition of a doubly ligated phycourobilin (PUB) at C50, 61 with an absorbance peak at 492 nm. Mutant variants CpeB-C50A (C50A; *orange line*), CpeB-C61A (C61A; *purple line*), and CpeB double mutant C50A/C61A (DM; *red line*) are shown. These samples were resolved by SDS-PAGE and analyzed by zinc-enhanced fluorescence of bound PUB excited at 488 nm and bound PEB excited at 532 nm (*B*–*C*). Western blot analysis using anti-CpeB antibodies was used to detect the total amount of CpeB present (*D*). This data is representative of two independent biological replicates.

Site-directed mutagenesis was used to check the hypothesis that the doubly ligated PUB at CpeB C50, 61 requires linkage along the A ring for the isomerization to occur. A control coexpression of CpeA/B, CpeZ, CpeS, and MpeV produced the greatest amount of C82-PEB and C50, 61-PUB chromophorylation and is denoted as the wild-type (WT) expression for this experiment. Two mutant variants of RS9916 CpeB C50 or C61 in which the cysteine residue was changed to alanine (C50A or C61A, respectively) were coexpressed with the lyases above. For the C50A mutant, we found that loss of linkage to the A ring significantly reduced the ability for a bilin to attach to the D ring *via* C61. Indeed, the C50A variant shows only a spectral signature for PEB ligation with no evidence of PUB ligation in the absorbance spectrum (Fig. 6*A*; orange line) or in the zinc-enhanced fluorescence (Fig. 6, *B*–*C*). For the C61A variant, absorbance at 495 nm (indicative of bound PUB) is detected, with a 3 nm red shift when compared with the WT (Fig. 6*A*; purple and black lines, respectively). This shift may be caused by the PUB being held in a less rigid conformation within the binding pocket due to the loss of addition along the D-ring (Fig. 6*A*; purple line). The double mutant (DM) C50A/C61A behaved as a negative control (Fig. 6*A*; red line; Fig. 6, *B*–*D*) for PUB addition in these studies and was not analyzed further. The near absence of zinc-enhanced PUB fluorescence at 488 nm for the C50A mutant (Fig. 6*B*) compared with the strong PEB fluorescence at 532 nm (Fig. 6*C*) matches the absorbance spectra results, indicating loss of PUB addition in this mutant, supporting our hypothesis that ring A addition is required for isomerization to occur. Western blot analysis using Anti-CpeB antibodies shows that CpeB is present in all samples (Fig. 6*D*). Ratios of band intensities of bound bilin using zinc-enhanced fluorescence of PEB (attached by CpeS) and PUB (attached by MpeV) and total substrate present (western blot detection of CpeB) were normalized to WT and reveal that 95.7 ± 2.6% of all available CpeB-C82 was modified by PEB for all mutant variants when compared with WT (Table 1). The C50A mutant recapitulated the negative control DM when band intensities were also normalized to WT. The small amount of PUB fluorescence detected when gels were excited at 480 nm is likely background, since C50A has a band intensity of 2.3% ± 1.2% while the DM shows a band intensity of 2.8% ± 0.3% when compared with WT (Fig. 6*B*). Loss of linkage at the D ring reduces the yield of PUB attached to CpeB by MpeV *via* linkage only at the A ring at the C50 residue (30.5% ± 19.5%; Table 1).

**Table 1:** Summary of recombinant CpeB mutant variants

Recombinant sample^a^ (abbreviation)	Cysteine(s)	Bilin attached	Percentage chromophorylation (% ± SD)^b^
CpeB+SVZ (WT)	82	PEB	100^c^
CpeB+SVZ (WT)	50, 61	PUB	100^c^
CpeBC50A+SVZ (C50A)	82	PEB	85.7 ± 10.0
CpeBC50A+SVZ (C50A)	50, 61	ND^d^	8.3 ± 7.2
CpeBC61A+SVZ (C61A)	82	PEB	81.7 ± 21.0
CpeBC61A+SVZ (C61A)	50, 61	PUB	49.8 ± 4.3
CpeBC50A/C61A+SVZ (DM)	82	PEB	89.0 ± 12.3
CpeBC50A/C61A+SVZ (DM)	50, 61	ND	ND

a All samples were expressed the presence of bilin synthesis genes and CpeA. S, *p*CpeS; V, *p*HTMpeV; Z, *p*HTCpeZ.

b These results are representative of two independent biological replicates. Calculations performed as stated in methods.

c Mutant variants normalized to WT as fully chromophorylated (100%).

d ND denotes not determined due to difficulty detecting bilin.

**Table 2:** Observed LC-MS-MS peaks of trypsin-digested recombinant PE peptides

Sample^a^	α-82	α-139	β-82^b^	β-165	β-50, 61^b^
A/B+S+PEB	ND^c^	Unmod^d^	PEB (91.1%)	Unmod	Unmod
A/B+V+PEB	Unmod	Unmod	Unmod	ND	Unmod
A/B+V+S+PEB	ND	Unmod	PEB (95.3%)	Unmod	PUB (31.3%)
A/B+ZS+PEB	ND	Unmod	PEB (92.1%)	Unmod	Unmod
A/B+V+ZS+PEB	ND	Unmod	PEB (98.2%)	Unmod	PUB (29.4%)


Sample	α-75	α-83	α-140	β-82	β-159	β-50, 61
MA/B+V+S+PEB	ND	ND	ND	PEB	ND	Unmod
MA/B+V+ZS+PEB	ND	ND	ND	PEB (99.7%)	ND	PUB (84.7%)

a A/B, *p*HTCpeA/HTCpeB; S, *p*CpeS; V, *p*HTMpeV; ZS, *p*HTCpeZ/CpeS; MA/B, *p*HTMpeA/HTMpeB.

b Parenthetical values represent calculated percentage of observed peptides modified by bilin as indicated. These results are representative of two independent biological replicates.

c ND represents peptides that were not detected.

d Unmod represents peptides that were unmodified and lack a bilin chromophore.

### LC-MS-MS analysis of recombinant RS9916 mutant variants

**Figure 7:**
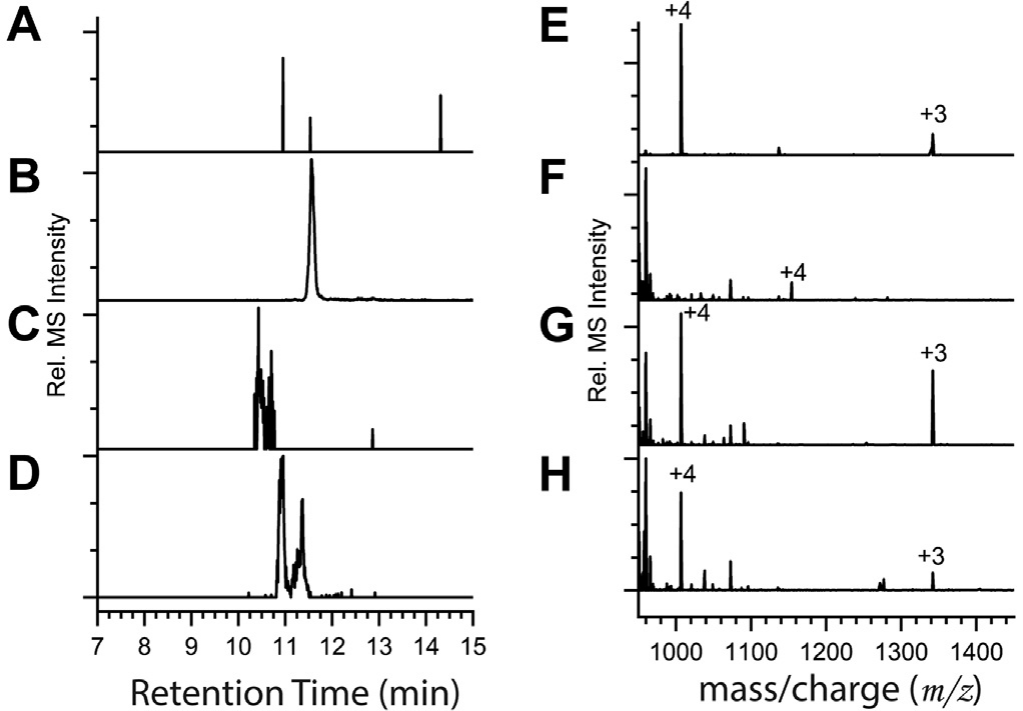
**Extracted ion chromatograms and LC-MS from recombinant RS9916 CpeB mutant coexpressions showing PUB bilin addition along C50, 61 residues.** Coexpressions and abbreviations are same as Figure 6. *A*, extracted ion chromatogram (EIC) of 1153.8063 (37–77) ^4+^ from C50A CpeB mutant. *B*, EIC for 1006.7336 (37–77 unmod) ^4+^ from C50A mutant. *C*, EIC for 1153.8063 from C61A mutant. *D*, EIC for 1006.7337 from C61A mutant. *E*, mass spectrum (MS) of 11.56 min peak in *B*. *F*, MS from 10.43 min peak in *C*. *G*, MS from 10.96 min peak in *D*. *H*, MS from 11.37 min peak in *D*. The observed *m/z* ratios for each of the labeled peaks are listed in Table 3. This data is representative of two independent biological replicates.

LC-MS-MS of the recombinant CpeB proteins reveals no peptide ions from the C50A mutant coexpressions modified with PUB at the C61 position as hypothesized (blank EIC in Fig. 7*A*) if the order of attachment was promiscuous. The peak with a retention time of 11.51 min (Fig. 7*B*) reveals ions at *m/z* 1006.73^+4^ and a tandem mass spectrum consistent with unmodified peptide 37 to 77 from the C50A CpeB mutant (Fig. 7*E* and S3, top; see also Table 3). The C61A mutant data shows a mix of modified and unmodified C50-containing peptide fragments (Table 3 and Fig. S3, middle, and Table S3). The EIC from C61A CpeB mutant for *m/z* 1153.8 (37–77-PUB)^4+^ reveals a peak with a retention time of 10.43 min, and its mass spectrum (MS) shows PUB bound to C50 (Fig. 7, *C* and *F* and Fig. S3, bottom, Table S2). Figure 7*D* shows the EIC for *m/z* 1006.73 from C61A mutant. Figure 7*G* shows the MS from 10.96 min peak in Figure 7*D*, and Figure 7*H* shows the MS from 11.37 min peak in Figure 7*D*. The observed *m/z* ratios for each of the labeled peaks are listed in Table 3. Fig. S3 is the tandem mass spectra for the peptide ions shown in Figure 7, *E*–*H*; Table S2 shows the masses and identities of the labeled ions in Fig. S3. None of the mutant variants show significant disruption of PEB addition to C82 of CpeB under any conditions (MSMS in Fig. S4, m/z ratios in Tables 1 and S3).

**Table 3:** Observed *m/z* for labeled peaks in Figure 7

Sample	Phycobilin binding sites: Cysteine (C), alanine (A)	Observed *m/z*	Bilin added^a^
C50A	A50, C61	1153.8063^4+^ 1006.7336^4+^	PUB (0%)^b^ Unmod^c^
	C82	417.5401^3+^, 625.8069^2+^ 370.6660^2+^	PEB (76.3%) Unmod^d^
C61A	C50, A61	1153.7832^4+^ 1006.9838^4+^, 1341.9752^3+^	PUB (7.6%) Unmod^c^
	C82	417.5401^3+^, 625.7944^2+^ 370.6660^2+^	PEB (100%) Unmod^b^

a Parenthetical values represent calculated percentage of observed peptides modified by bilin indicated when compared with unmodified peptides. For sample abbreviations, see legend for Figure 6. These results are representative of two independent biological replicates.

b Not detected.

c Unmod represents peptides that were unmodified and lack a bilin chromophore.

d Cys modified by β-mercaptoethanol.

## Discussion

In this study, we demonstrate that the *mpeV* gene of the marine *Synechococcus* strain RS9916 encodes the lyase isomerase responsible for the addition of PEB and simultaneous isomerization to PUB at the doubly linked Cys-50, 61 position of the β-subunits of both PEI and PEII. Recombinant protein expression in *E. coli* of MpeV alone proved to be insufficient to chromophorylate either CpeB or MpeB (Fig. 4). A prior ligation of PEB by CpeS at the C82 position to CpeB and MpeB, respectively, was necessary to stabilize the PEI and PEII β-subunits and provide MpeV access to the folded substrate. These results are consistent with previous studies on the freshwater cyanobacteria *F. diplosiphon* and *Synechococcus* sp. PCC 7002, showing that lack of bilins at the central C82 equivalent position affects the stability and turnover rates of PBPs and reduces their folding and solubility in *E. coli* (10, 17, 21, 46, 51, 52). Functionality and efficiency of some lyases are also known to depend upon substrate association with important chaperone-like proteins (14, 30, 34, 53). Here, the chaperone-like protein CpeZ from RS9916, which is orthologous to the characterized CpeZ from *F. diplosiphon*, was shown to enhance the chromophorylation of CpeB and MpeB by MpeV and CpeS, presumably by stabilizing the β-subunits, preventing their aggregation, so that lyases could act (Fig. 4*A*). When CpeS was the sole lyase in the expression vector, the presence of CpeZ slightly increased the addition of PEB (Fig. 4*C*); however, when coexpression also included MpeV, CpeZ played a much larger role in assisting in chromophorylation (Fig. 4). Of note, very little soluble substrate was detected in any sample containing only CpeZ and/or MpeV when compared with controls. This suggests that CpeZ has a role in maintaining substrate (*e.g.*, CpeB or MpeB) stability in RS9916, perhaps by preventing aggregation, allowing time for lyases to act. Once chromophorylated, CpeB is much more soluble.

A phylogenetic analysis of the protein family of the lyase isomerase MpeV showed that it likely derives from a freshwater CpeF-like PEB lyase ancestor (Fig. 3). Although MpeV is not directly involved in CA4 since CpeB and MpeB do not change chromophorylation between blue and green light [27], it is worth noting that this enzyme forms a well-defined subfamily that is specifically found in CA4-A strains, one of the two *Synechococcus* CA types (with CA4-B), while its paralog MpeU is found in a much larger set of pigment types, encompassing both CA4 types and the two known blue light specialists (3c and 3f; Fig. 3). MpeU is also a lyase isomerase, but its substrate specificity remains unknown [38]. Advances made in the present study about the necessity of coexpressing *cpeS* and *cpeZ* with the lyase to be characterized may help unveil MpeU substrate specificity in the near future. Also, refined comparative analyses of lyases and lyases isomerases of this protein family should help identify residues involved in isomerase activity in MpeU and MpeV.

No study to date has assessed the importance of prior bilin ligation and thioether formation on the isomerization capacity of lyase isomerases. Lyases are required to orient and attach bilin to their appropriate substrate, avoiding erroneous or improper attachment and allowing for maximized energy transfer through the PBS (4, 14, 22, 54). Using mutant variants of CpeB, we found that mutating the C50 residue eliminated the ability of MpeV to covalently attach PUB to CpeB (Fig. 6 and Table 1). This is likely due to bilin addition primarily occurring along the A-ring (to C50) followed by subsequent attachment along the D ring (to C61). Recent studies suggest that double attachment of a bilin is contingent on linkage at the A ring prior to linkage at the D ring rather than the reverse (30, 46). Conversion of PEB to PUB involves isomerization of a double bond from the C^4^-C^5^ carbons to the C^2^-C^3^ carbons, located on the A ring. We hypothesized that loss of A ring linkage inhibits isomerization of PEB to PUB resulting in singly attached PEB (*via* D ring) to C61 in our CpeB-C50A mutant variant. However, we saw a complete loss of bilin addition to C61 as determined by LC-MS-MS (Tables 1 and 3, and Fig. 7). In RS9916, it appears that linkage along the A ring is the crucial first step in adding PUB across C50 and C61 on the β-subunits. It is possible that MpeV binds and isomerizes PEB prior to ligation, or perhaps the isomerization is occurring once both PEB and MpeV are interacting with the binding pocket even if ligation is not taking place. The C61A mutant results show that loss of C61 as a ligation site does not affect the ability of MpeV to attach PEB and isomerize it to PUB at C50 (Fig. 7). A red spectral shift of 3 nm for the PUB absorbance peak suggests that the bilin, though ligated, is not equivalently held within the pocket. Since the C50, C61 binding pocket is located along the exterior of CpeB (Fig. S5), having a doubly ligated bilin appears to be necessary to maintain bilin stability and increase light capture and energy transfer efficiency. Mutation of C50 and/or C61 had no effect on PEB ligation to C82 *via* CpeS in any of our mutant variants (Table 1), consistent with the idea that CpeS must act first and this PEB at C82 stabilizes the CpeB subunit.

Continued characterization of these lyases and lyase isomerases is an important step in understanding the biosynthesis of the PBS and how these globally important primary producers are able to grow and thrive in the changing light environments of the open ocean.

## Experimental procedures

### Phylogenetic comparisons

A Bayesian tree was generated for the MpeU, MpeV, MpeX, and CpeF protein families. MpeU, MpeV, MpeX, and CpeF protein sequences were retrieved either from Genbank or from the Cyanorak v2.1 database (www.sb-roscoff/cyanorak) for *Prochlorococcus* and *Synechococcus* genomes (Fig. 3 and Table S4). Phylogenetic reconstructions were performed using Maximum Likelihood (ML; PhyML v3.0) and Bayesian Inference (BI; MrBayes v3.1.2). Likelihood scores of 120 potential evolutionary models were evaluated using the Akaike Information Criteria (AIC) (55) and Bayesian Information criteria (BIC) (56), as implemented in Protest 3.4.1 (57). ML reconstructions were performed using PhyML (v3.0) (58) with the Le and Gascuel substitution model, with estimation of the Γ distribution parameter, fraction of invariant sites, and character frequencies (LG+I + F + G model) (59) and using 100 bootstrap replicates. Bayesian inference was done by invoking the appropriate “nst” and “rates” settings in the software package MrBayes 3.1.2 (v3.2.1) (60) using the same model. The Markov Chain Monte Carlo (MCMC) analysis algorithm was used to estimate the posterior probability distribution for each collection of sequences using one incrementally “heated” chain with three “cold” chains, these four chains being replicated four times per analysis, a random starting tree, and sampling every 100th generation. Bayesian posterior probabilities were generated from 5,000,000 generations, and the first 12,500 generations were removed as burn-in. All reconstructions were visualized using Archaeopteryx Version 0.9901 (61), and the tree was drawn using iTOL (62).

### Cloning of *Synechococcus* genes

The putative lyase genes *cpeS*, *mpeV*, and *cpeZ* from *Synechococcus* sp. RS9916 genome (Fig. S2) were amplified *via* polymerase chain reaction (PCR) using *Pfu* DNA polymerase. Amplified fragments were separately cloned into compatible Novagen Duet vectors using corresponding restriction enzymes as listed in Tables S5 and S6. Expression vectors used in this study (Table S6) include three previously described (46). The RS9916 genes *mpeA*, *cpeA*, *cpeB*, and *mpeB* sequences were inserted into multiple cloning site I (MCSI) pET-Duet (Novagen, Madison, WI) in frame with the sequence encoding a hexahistidine tag (HT) at the amino terminus. *cpeB* and *mpeB* were subsequently subcloned in to MCSII to achieve (MCSI/MCSII/vector) RS9916 HT*cpeA*/HT*cpeB/p*ET-DUET and RS9916 HT*mpeA*/HT*mpeB/p*ET-DUET (Table S5). RS9916 *cpeZ* was cloned using Platinum SuperFi PCR protocol (ThermoFisher Scientific, Waltham, MA) and inserted into MCSI of pCDF-Duet in frame with the sequence encoding a HT at the amino terminus (Tables S5 and S6).

### Analysis of recombinant protein and bound bilin

*In vivo* heterologous protein expression was performed under reduced ambient light conditions using *E. coli* as previously described (21) with the following modifications: a 100 ml starter culture of recombinant plasmid containing *E. coli* cells was grown at 37 °C overnight, then added to 1 L of Luria Bertani growth media at 18 °C. Once an OD = 0.6 was achieved, cultures were induced and maintained at 18 °C for 24 h with shaking. Cells were collected by centrifugation at 11,000*g* for 8 min. The wet weight of all cell pellets (averaging from 4.77 to 5.74 ± 0.41 g) was measured and recorded prior to storage at −20 °C. Cell pellets were resuspended at 3.0 ml·g^−1^ in equilibration buffer (20 mM sodium phosphate, 300 mM sodium chloride with 10 mM imidazole, pH 7.4) supplemented with mini protease cocktail (Thermo Scientific, Waltham, MA) and 0.01 mg·ml^−1^ lysozyme (Fisher Scientific, Hampton, NH). Table S1 summarizes the combinations of lyases tested. Proteins were purified using Ni-affinity column chromatography (Thermo Scientific, Rockford, IL), washed four times with 10 ml wash buffer (20 mM sodium phosphate, 300 mM sodium chloride with 25 mM imidazole, pH 7.4), and collected with 12 to 15 ml of elution buffer (20 mM sodium phosphate, 300 mM sodium chloride with 250 mM imidazole, pH 7.4). Eluents were dialyzed against 20 mM Tris HCl 100 mM Na/KCl (pH 8.0) and 1 mM β-mercaptoethanol at 4 °C. Samples were concentrated by ultrafiltration through an Amicon Ultra centrifugal filter unit (10 kDa cutoff; Novagen/EMD Millipore Corp, Darmstadt, Germany). Protein content was quantified using Bradford colorimetric assay (BioRad, Hercules, CA) and diluted to obtain equal concentrations of total protein. Absorbance spectroscopy was performed using Perkin Elmer Lambda 35 UV/VIS spectrophotometer followed by fluorescence spectroscopy using a Perkin Elmer LS 55 Fluorescence Spectrometer (Waltham, MA) with excitation at 490 nm (PEB) or 440 nm (PUB) and emission at 570 nm for a range of 400 to 750 nm (slit widths were set at 10 nm). Proteins were subsequently resolved by 15% (w/v) polyacrylamide gel electrophoresis (PAGE) in the presence of sodium dodecyl sulfate (SDS) and ultimately visualized by Coomassie blue staining (10). To visualize proteins with bound bilin, gels were subjected to zinc-enhanced fluorescence prior to Coomassie blue staining using ChemiDoc MP imaging system (Bio-Rad, Hercules, CA) with excitation at 488 nm (PUB) and 532 nm (PEB).

### Western blot analysis of CpeB proteins

Western blotting was performed using the Trans-Blot Turbo rapid transfer system (BioRad Hercules, CA). Two identical SDS-polyacrylamide gels were loaded with prestained molecular weight standards (Bio-Rad, Hercules, CA) and samples. The control gel was stained with ZnSO_4_ followed by Coomassie blue while the other gel was transferred to PVDF (polyvinylidene difluoride) membrane for Western blot detection, using the Trans-Blot Turbo mini 0.2 μm PVDF transfer pack. Proteins were blotted at 1.3 amps and 25 V for 7 min. After transfer, membranes were placed in blocking buffer (TBST [20 mM Tris, pH 7.6, 137 mM NaCl, and 0.1% (v/v) Tween-20] and 5% (w/v) nonfat dried milk) for 12 h at 4 °C and then washed with TBST. Membranes were then incubated in 40 ml TBST with the primary polyclonal rabbit antibody Anti-CpeB #YZ5017 at a 1:40,000 dilution for 1 h (YenZym Antibodies, San Francisco, CA). Anti-CpeB antibodies were generated against holo-CpeB purified from *F. diplosiphon* (15). Membranes were further washed and incubated with secondary antibodies as previously described (46). Luminal/enhancer and peroxide reagents (Bio-Rad, Hercules, CA) for enhanced chemiluminescence were mixed in a 1:1 ratio and incubated with the membrane for 1 min. Chemiluminescence was detected by a Chemi-Doc MP Imaging System (Bio-Rad, Hercules, CA). Membranes were stored in TBST at 4 °C. Protein volume intensities were quantified and analyzed using Image Lab Software V5.2.1 (Bio-Rad, Hercules, CA).

### Site-directed mutagenesis and analysis of chromophorylation and isomerization

Site-directed mutants were created for RS9916 CpeB using combined overlap extension PCR method adapted from (63) with the modification that Platinum SuperFi (Thermo) enzyme was used for all PCR reactions. Oligonucleotide primers generated for site-directed mutants are listed in Table S7. Two single-site mutant variants of RS9916 CpeB were created by mutating the C50 residue to alanine (C50A) and the C61 residue also to alanine (C61A). PCR fragments were cloned into MCSII of pET-DUET vector containing HTCpeA in MCSI with resulting plasmids listed in Table S7. A double mutant C50A/C61A was created by performing combined overlapping PCR with the C50A mutant as the template for C61A mutagenesis. Using ChemiDoc MP imaging system (Bio-Rad, Hercules, CA), intensity of bands excited at 488 nm (PUB) and 532 nm (PEB) was compared with the intensity of bands from western blots to achieve an estimation of bilin chromophore prevalence on available substrate when compared to a control coexpression (Table 1).

### Trypsin digestion and liquid chromatography tandem mass spectrometry

Purified proteins were dialyzed against 2 mM sodium phosphate buffer (pH 7.0) and 1 mM β-mercaptoethanol. One aliquot of trypsin (dimethylated trypsin from porcine pancreas; Sigma, St Louis, MO) was added to 2% (w/w) from a 20 μg ml^−1^ stock to the denatured protein mixtures and incubated at 30 °C for 3 h in the dark (31). The reaction was quenched by adding 30% (v/v) glacial acetic acid. Digested peptides were passed through a pre-equilibrated C8 Sep-Pak cartridge (Waters Corporation, Milford, MA), thereafter the eluted sample was vacuum dried and stored at −80 °C before LC-MS-MS (LC-MS^2^) analysis on a Thermo Orbitrap Fusion Lumos instrument using 120,000 resolving power precursor ion scans (m/z 250–1500) with data-dependent HCD (3 s cycle time, quadrupole precursor isolation window = 2 m/z, 30,000 resolving power product ion scans, 30% relative collision energy, 5% steps). Samples were separated by an Agilent 1100 CapLC with a 0.3 × 100 mm Zorbax SB300 C18 column using conditions described in Kronfel *et al*. (34). All data processing was performed with Thermo XCalibur 4.0, Proteome Discover 2.1.1.21 (Thermo), and a local copy of ProteinPropector 5.22.1 (prospector.ucsf.edu) (64). The FASTA file searched had the seven recombinant protein sequences (Table S1), and the following parameters were used to generate the *in silico* peptide library. Trypsin digestion, SEQUEST search engine, 10 ppm precursor tolerance, 0.02 Da fragment mass tolerance, two missed cleavage sites per peptide, a, b, and y ions considered, up to four modifications were allowed to a single peptide including oxidized methionine (+O), mercaptoethanol addition to Cys (+C_2_H_4_OS), bilin addition to Cys (+C_33_H_38_N_2_O_6_), oxidized bilin addition to Cys (+C_33_H_38_N_2_O_7_), and deamidation of Gln or Asn (-NH, +O), and loss of NH_3_ from peptide N-terminal Asn residues, loss of protein N-terminal Met, or acetylation of N-terminus. Default parameters for estimating identification confidence with XCorr values were used and varied by charge state (>1.9 for 2+, 2.3 for 3+, and 2.6 for 4+ EICs were extracted with ±4 ppm windows around the predicted ion *m/z* ratios). MS quantitation used raw EIC peak areas for all species without any external standards; the peak areas from multiple charge states of the same peptides were combined during integration.

## Data availability

All biochemistry experimental data are contained within the article. The mass spectra raw files and search output are stored on the MassIVE database at https://doi.org/10.25345/C5TV1Z.

## Conflict of interest

The authors declare that they have no conflicts of interest with the contents of this article.
