# Supplementary material for: MpeV is a lyase isomerase that ligates a doubly linked phycourobilin on the β-subunit of phycoerythrin I and II in marine Synechococcus
Source: J Biol Chem. 2020 Nov 23;296:100031. doi: 10.1074/jbc.RA120.015289 (PMC7948978; doi:10.1074/jbc.RA120.015289)
Supplement: Supplementary Figures and Tables [file mmc1.pdf]

Supporting Information

MpeV is a lyase isomerase that ligates a doubly-linked phycourobilin on the β-subunit of phycoerythrin I and II in marine *Synechococcus*

Lyndsay A. Carrigee[1], Jacob P. Frick[1], Jonathan A. Karty[2], Laurence Garczarek[3], Frédéric Partensky[3], and Wendy M. Schluchter[1*]

Table S1: RS9916 recombinant protein co-expressions and abbreviations
Table S2: Masses of peaks labeled in Fig. S3
Table S3: Masses of peaks labeled in Fig. S4
Table S4: Accession numbers of the lyase sequences used in this study for phylogenetic analyses
Table S5: Oligonucleotide primers used in the cloning of *Synechococcus* genes
Table S6: Plasmids used in this study
Table S7: *Synechococcus* sp. RS9916 site directed mutagenesis of CpeB
Figure S1: Predicted structure and amino acid sequence comparison of MpeV protein from *Synechococcus* sp. RS9916 and CpeF protein from *Fremyella diplosiphon.*
Figure S2: Map of the central part of the phycobilisome rod genomic region of *Synechococcus* sp. RS9916
Figure S3: Tandem mass spectra for CpeB mutant co-expressions
Figure S4: Extracted ion chromatograms and LC-MS from recombinant RS9916 CpeB mutant co-expressions showing PEB bilin addition to C82 residue
Figure S5: Bilin structure and ligation of RS9916 CpeB

**Table S1.** RS9916 recombinant protein co-expressions and abbreviations.		
**PE subunit[a]**	**Lyase[b]**	**Recombinant co-expression abbreviations[c]**
HTCpeA/HTCpeB	--	CpeAB
HTCpeA/HTCpeB	CpeS	CpeAB+CpeS
HTCpeA/HTCpeB	CpeZ	CpeAB+CpeZ
HTCpeA/HTCpeB	MpeV	CpeAB+MpeV
HTCpeA/HTCpeB	CpeS+CpeZ	CpeAB+CpeZS
HTCpeA/HTCpeB	CpeS+MpeV	CpeAB+CpeS+MpeV
HTCpeA/HTCpeB	MpeV+CpeZ	CpeAB+MpeV+CpeZ
HTCpeA/HTCpeB	MpeV+CpeS+CpeZ	CpeAB+CpeZS+MpeV
HTMpeA/HTMpeB	--	MpeAB
HTMpeA/HTMpeB	CpeS	MpeAB+CpeS
HTMpeA/HTMpeB	CpeZ	MpeAB+CpeZ
HTMpeA/HTMpeB	MpeV	MpeAB+MpeV
HTMpeA/HTMpeB	CpeS+CpeZ	MpeAB+CpeZS
HTMpeA/HTMpeB	CpeS+MpeV	MpeAB+CpeS+MpeV
HTMpeA/HTMpeB	MpeV+CpeZ	MpeAB+MpeV+CpeZ
HTMpeA/HTMpeB	MpeV+CpeS+CpeZ	MpeAB+CpeZS+MpeV

[a]HT indicates the presence of a hexa-histidine tag fused to protein product
[b]Lyase enzymes expressed from clones of RS9916 genes of same name
[c]Expressed with PebS/HoI to generate PEB from *E. coli'*s heme

Table S2. Masses of peaks labeled in Fig. S3. [1]Formulas provided for bilin-containing fragments. [2]Ions are isobaric and cannot be distinguished by MS-MS.

			C50A no bilin (S3 top)			C61A no bilin (S3 middle)			C61A bilin (S3 bottom)		
ion	formula[1]	theo. m/z	m/z obs	Δ in mTh	Δ in ppm	m/z obs.	Δ in mTh	Δ in ppm	m/z obs.	Δ in mTh	Δ in ppm
y1		175.1190	175.1188	-0.2	-1.1	175.1189	-0.1	-0.6	175.1191	0.1	0.6
b2		229.1183	229.1182	-0.1	-0.4	229.1182	-0.1	-0.4	229.1182	-0.1	-0.4
b3		300.1554	300.1553	-0.1	-0.3	300.155	-0.4	-1.3	300.1553	-0.1	-0.3
bilin BC rings	$C_{19}H_{23}N_2O_4^+$	343.1652	N/A			N/A			343.1649	-0.3	-0.9
y3		386.2146	386.2141	-0.5	-1.3	386.2145	-0.1	-0.3	386.2147	0.1	0.3
b4		399.2238	399.2234	-0.4	-1.0	399.2232	-0.6	-1.5	399.2234	-0.4	-1.0
bilin ABC or BCD rings[2]	$C_{26}H_{30}N_3O_5^+$	464.2180	N/A			N/A			464.2175	-0.5	-1.1
b5		513.2667	513.2668	0.1	0.2	513.2659	-0.8	-1.6	513.2673	0.6	1.2
y4		549.2780	549.2769	-1.1	-2.0	549.2771	-0.9	-1.6	549.2773	-0.7	-1.3
b6		584.3039	584.3033	-0.6	-1.0	584.3031	-0.8	-1.4	584.3036	-0.3	-0.5
Free bilin fragment	$C_{33}H_{39}N_4O_6S^+$	587.2864	N/A			N/A			587.2869	0.5	0.8
b7		697.3879	697.3886	0.7	1.0	697.3868	-1.1	-1.6	697.3872	-0.7	-1.0
b8		798.4356	798.4377	2.1	2.6	798.4346	-1.0	-1.3	798.4355	-0.1	-0.1
y8		880.3730	Not Observed			Not Observed			880.3716	-1.4	-1.6
y9		951.4101	Not Observed			Not Observed			951.4045	-5.6	-5.9
b11		1070.5477	1070.5482	0.5	0.5	1070.555	6.9	6.4	Not Observed		
y10		1079.4687	Not Observed			Not Observed			1079.4692	0.5	0.5
y11		1192.5528	Not Observed			Not Observed			1192.5481	-4.7	-3.9
y12		1305.6368	Not Observed			Not Observed			1305.6235	-13.3	-10.2
y13		1362.6583	Not Observed			Not Observed			1362.6475	-10.8	-7.9
y14		1463.7060	Not Observed			Not Observed			1463.6992	-6.8	-4.6
y17		1777.8286	Not Observed			Not Observed			1777.8208	-7.8	-4.4

Table S3. Masses of peaks labeled in Fig. S4. [1]Formulas provided for bilin-containing fragments.

ion	formula[1]	theo. m/z	m/z obs	Δ in mTh	Δ in ppm	m/z obs.	Δ in mTh	Δ in ppm
a2		175.0900	175.0900	0.0	0.0	175.0900	0.0	0.0
y1		175.1190	175.1189	-0.1	-0.6	175.1190	0.0	0.0
b2		203.0849	203.0848	-0.1	-0.5	203.0848	-0.1	-0.5
y2		288.2030	288.2029	-0.1	-0.3	288.2028	-0.2	-0.7
bilin CD ring	$C_{17}H_{19}N_2O_3^+$	299.1390	299.1389	-0.1	-0.4	299.1389	-0.1	-0.4
bilin BCD ring	$C_{25}H_{28}N_3O_5^+$	450.2024	450.2021	-0.3	-0.6	450.2019	-0.5	-1.0
$y4+ABCring^{2+}$	$C_{44}H_{66}N_{10}O_{10}S^{2+}$	463.2337	463.2333	-0.4	-0.9	463.2332	-0.5	-1.1
bilin ABC ring	$C_{26}H_{32}N_3O_5^+$	466.2337	466.2328	-0.9	-1.8	466.2328	-0.9	-1.8
$y5+ABCring^{2+}$	$C_{47}H_{71}N_{11}O_{11}S^{2+}$	498.7522	498.7520	-0.2	-0.4	498.7520	-0.2	-0.4
y5 no bilin		533.2864	533.2858	-0.6	-1.1	533.2856	-0.8	-1.5
$y5+bilin^{2+}$	$C_{54}H_{80}N_{12}O_{12}S_2^+$	560.2864	560.2856	-0.8	-1.5	560.2853	-1.1	-2.0
Free bilin fragment	$C_{33}H_{39}N_4O_6S^+$	587.2864	587.2874	1.0	1.7	587.2872	0.8	1.3
peptide+H no bilin		664.3269	664.3277	0.8	1.2	664.3281	1.2	1.8
$Peptide+bilin^{2+}$		625.8067	625.8067	0.0	0.0	625.8072	0.5	0.8
$Peptide+bilin^{3+}$		417.5402	417.5400	-0.2	-0.5	417.5403	0.1	0.2

Table S4. Accession numbers of the lyase sequences used in this study for phylogenetic analyses.

- ⇨ See Excel table.

**Table S5.** Oligonucleotide primers used in the cloning of *Synechococcus* genes		
**RS9916**	HTcpeZ.F.BamHI	5’-GCATGGGATCCTGAGACTTTCTCGACGC-3’
**RS9916**	HTcpeZ.R.EcoRI	5’-GCATGAATTCGATGGACTCTTCTGTGCAATCG-3’
**RS9916**	cpeS.F.NdeI	5’-TATCGCTCATATGAATATTGAGCAATTTGTTGC -3’
**RS9916**	cpeS.R.XhoI	5’-TGTCTCGAGTTATGCGCTGATTCTTTTGACC-3’
**RS9916**	HTmpeV.F.EcoRI	5’- GCCGGAATTCGGAGATTCGAGAGAACACTC-3’
**RS9916**	HTmpeV.R.NotI	5’-ATATGCGGCCGCCTAGCCACCCTCAG-3’
**RS9916**	HTcpeA.F.BamHI	5’-GCATGGATCCGATGAAGTCTGTCGTGACCAC-3’
**RS9916**	HTcpeA.R.HindIII	5’-GCATAAGCTTTCAAGAAAGAGCGTTGATCA-3’
**RS9916**	HTcpeB.F.BamHI	5’-TCTGGATCCGATGCTCGACGCATTCTCCCGTTCGG-3’
**RS9916**	HTcpeB.R.SalI	5’-TGTGTCGACTCAGGAGACGGCTCCGATCACGCG-3’
**RS9916**	HTMpeA.F.BamHI (1)	5’-TCTCCCTTATGCGACTCCTGCATT- 3’
**RS9916**	HTMpeA.R.EcoRI (1)	5’-TGCGGCCGTGTACAATACGATTAC-3’
**RS9916**	HTMpeB.F.BamHI (1)	5’-TCTCCCTTATGCGACTCCTGCATT- 3’
**RS9916**	HTMpeB.R.EcoRI (1)	5’-TGCGGCCGTGTACAATACGATTAC-3’

**Table S6.** Plasmids used in this study				
**Plasmid name**[a]	**Recombinant proteins produced**[b]	**Parent vector**	**Antibiotic**[c]	**Reference**
pHTCpeA/HTCpeB	*Synechococcus* sp. RS9916 HT-CpeA/HT-CpeB	pETDuet-1	Ap	This study
pHTMpeA/HTMpeB	*Synechococcus* sp. RS9916 HT-MpeA/HT-MpeB	pETDuet-1	Ap	This study
pHTCpeZ	*Synechococcus* sp. RS9916 HT-CpeZ	pCOLADuet-1	Km	This study
pCpeS	*Synechococcus* sp. RS9916 NT CpeS	pCOLADuet-1	Km	This study
pHTCpeZ/CpeS	*Synechococcus* sp. RS9916 HT-CpeZ/NT CpeS	pCOLADuet-1	Km	This study
pHTMpeV	*Synechococcus* sp. RS9916 HT-MpeV	pCDFDuet-1	Sp	This study
pPebS	Myovirus Ho1 and NT-PebS	pACYCDuet-1	Cm	(2,3)

[a] Genes encoding hexahistidine-tags fused to protein product are indicated as "HT-"
[b] "NT-" is an abbreviation for "Non-Tagged" referring specifically to hexahistidine-tags
[c] Antibiotic resistance used to select for the presence of the plasmid (Ap: ampicillin; Cm: chloramphenicol; Km: kanamycin; Sp: spectinomycin)

**Table S7.** *Synechococcus* sp. RS9916 site directed mutagenesis of CpeB[+].		
**Primer Name**[*]	**Sequence (5' to 3')**[+]	**Plasmid Name**[a b]
CpeB.GSP.F.BamHI	TCTGGATCCGATGCTCGACGCATTCTCCCGTTCGG	pHTCpeA/HTCpeB
CpeB.GSP.R.SalI	TGTGTCGACTCAGGAGACGGCTCCGATCACGCG	
CpeB.MPC50A.F.BamHI	GCATCAACGCCTCCGCCATCGTTTCTGATG	pHTCpeA/ HTCpeBC50A
CpeB.MPC50A.R.HindIII	GAGTCATCAGAAACGATGGCGGAGGCGTTG	
CpeB.MPC61A.F.BamHI	GACTCGGCATGATCGCCGAGAACACCGGCC	pHTCpeA/ HTCpeBC61A
CpeB.MPC61A.R.HindIII	GCATGGTGTTCTCGGCGATCATGCC	
CpeBC50A.MPC61A.F.BamHI	GCATCAACGCCTCCGCCATCGTTTCTGATG	pHTCpeA/ HTCpeBC50,61A
CpeBC50A.MPC61A,R.HindIII	GCATGGTGTTCTCGGCGATCATGCC	

[+]Site-directed mutation in orange
[*]GSP denotes gene specific primer and MP denotes mutant primer
[a] All mutants were cloned into MCSI of *p*ET-DUET1 vectors containing non-mutated HT*cpeA* in MCSII with an antibiotic resistance to ampicillin (AP) -"
[b] Genes encoding hexahistidine-tags fused to protein product are indicated as HT

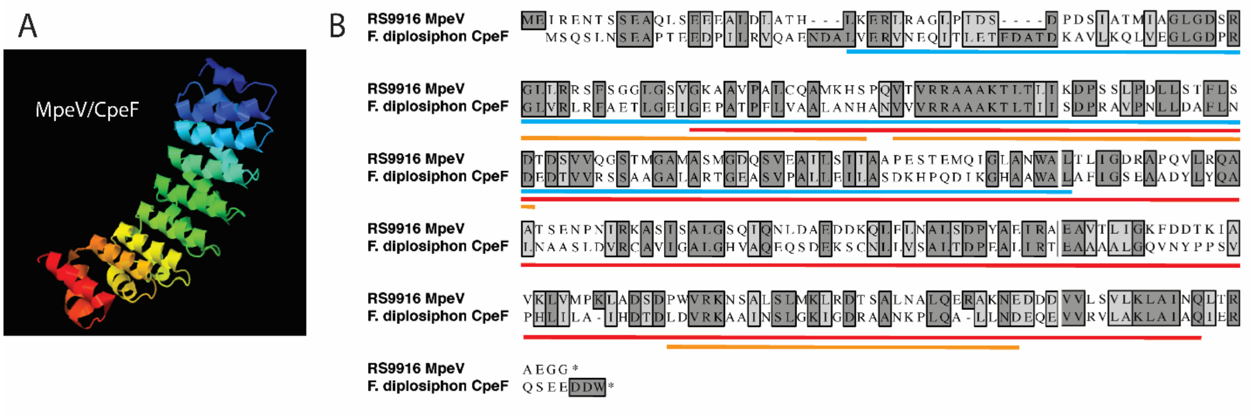

Figure S1. Predicted structure and amino acid sequence comparison of MpeV protein from *Synechococcus* sp. RS9916 and CpeF protein from *Fremyella diplosiphon.* **A.** Depiction of Phyre[2] predicted structure of orthologous lyase/lyase isomerase proteins from RS9916 MpeV and *F. diplosiphon* CpeF using the closest characterized structure PDB: bgfp-a (4). Color scheme follows red (N-terminus) to blue (C-terminus). **B.** Clustal W comparison of the amino acid sequences of CpeF and MpeV; identities are indicated with dark shading and similarities with light shading. Armadillo-like helical sequence (IPR011989: residues 68-290) is underlined in blue; Armadillo-type fold (IPR016024: residues 25-209) is underlined in red; PBS lyase HEAT-like repeat sequences (IPR004155: residues 54-82, 85-114, and 246-275) are underlined in orange.

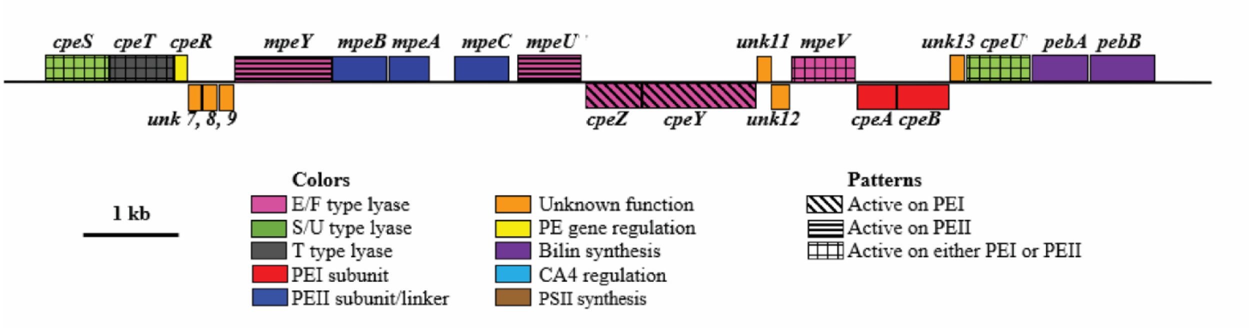

Figure S2. Map of the central part of the phycobilisome rod genomic region of *Synechococcus* sp. RS9916. This physical map includes the *cpeBA* (red) and *mpeAB* (dark blue) operons encoding the PEI and PEII subunits, respectively, all phycoerythrin-related lyase genes, one PEII linker and a number of genes of yet unknown function (5). Note that lyase genes present in the subregion extending from *unk7* to *mpeU* are specific to PEII, while those present in the rest of the region are either specific of PEI or can act on both PEI and PEII (1,5,6).

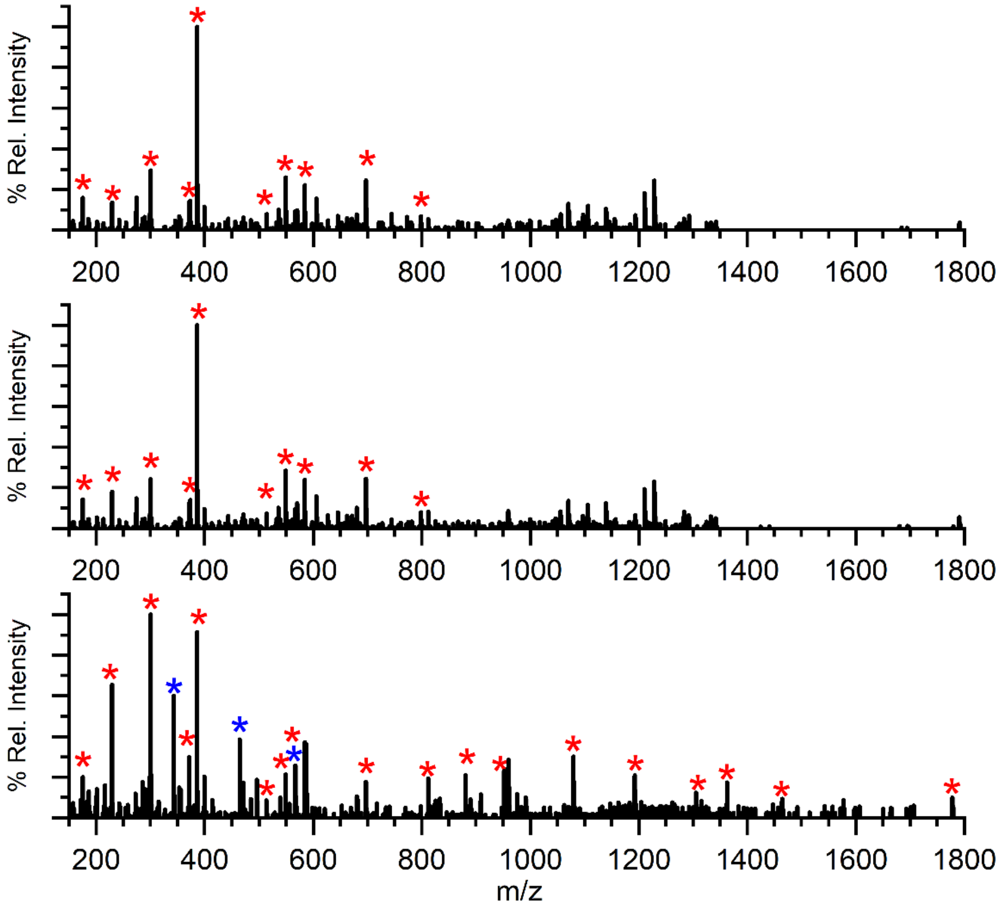

Figure S3. Tandem mass spectra for CpeB mutant co-expressions. Tandem mass spectra for (top) *m/z* 1006.23^{4+} from C50A mutant, (middle) *m/z* 1006.23^{4+} from C61A mutant (retention time = 11.39 min), and (bottom) *m/z* 1153.3061^{4+} from C61A. The peptide's sequence is LDAVNAITSNASC$^{\&}$IVSDAVTGMIAENTGLIQAGGNCYPNR, where $^{\&}$ indicates the location of bilin attachment in the bottom mass spectrum. Red * in the Figure indicate b and y ions identified for each mass spectrum; blue * indicate fragments containing parts of the bilin. The masses of all the observed fragments are compiled in Table S8.

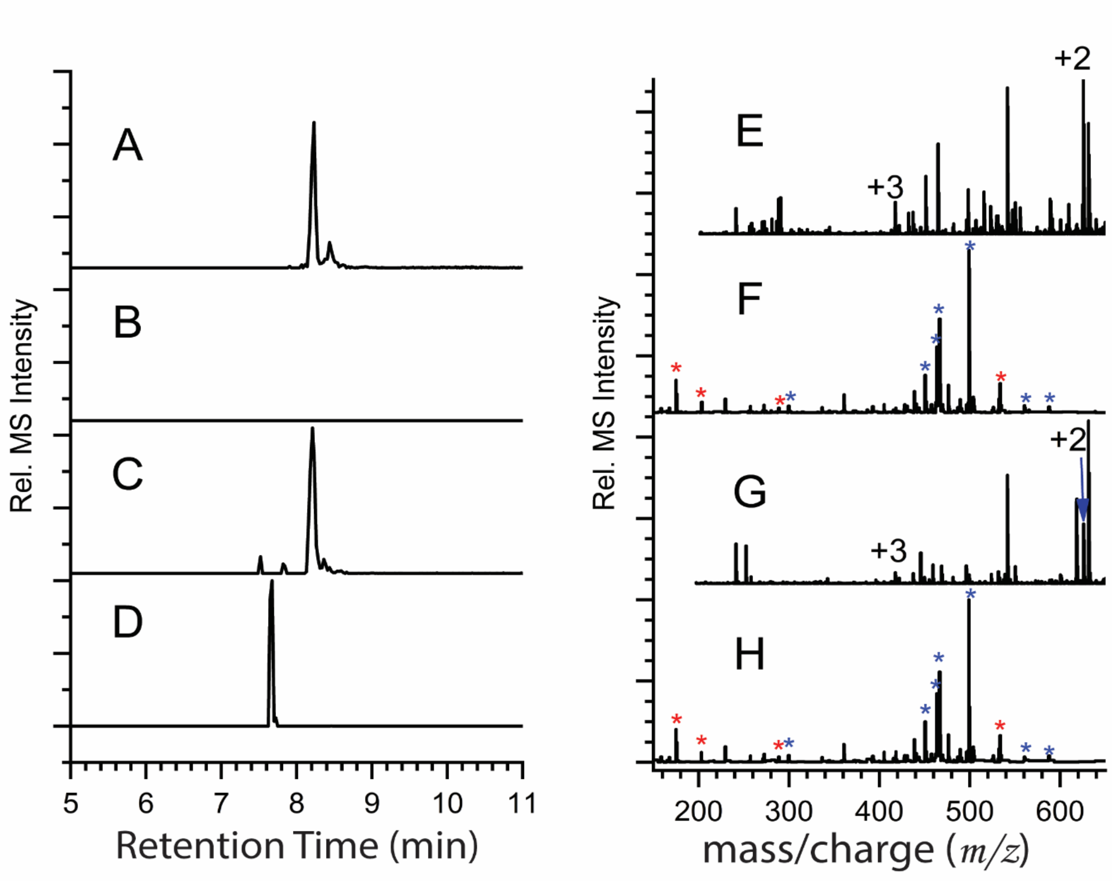

Figure S4. Extracted ion chromatograms and LC-MS from recombinant RS9916 CpeB mutant co-expressions showing PEB bilin addition to C82 residue. Extracted ion chromatograms (EICs) for **A**. *m/z* 417.5402 $(MAACLR+PEB)^{3+}$ from C50A mutant; **B**. *m/z* 370.6660 $(MAACLR+BME)^{2+}$ from C50A; **C**. *m/z* 417.5402 from C61A mutant; **D**. *m/z* 370.6660 from C61A. Mass spectra and tandem mass spectra from **E**. MS1 from 8.23 min peak in **A**; **B**. MS2 of 417.5400 peak in **E**; **G**. MS1 from 8.21 min peak in **C**; **H**. MS2 of 417.5403 peak in **G** (+3). Ions labeled with red * are fragments that do not contain any bilin or part of a bilin; ions labeled in blue contain all or part of a bilin as part of their composition (See also Table 3).

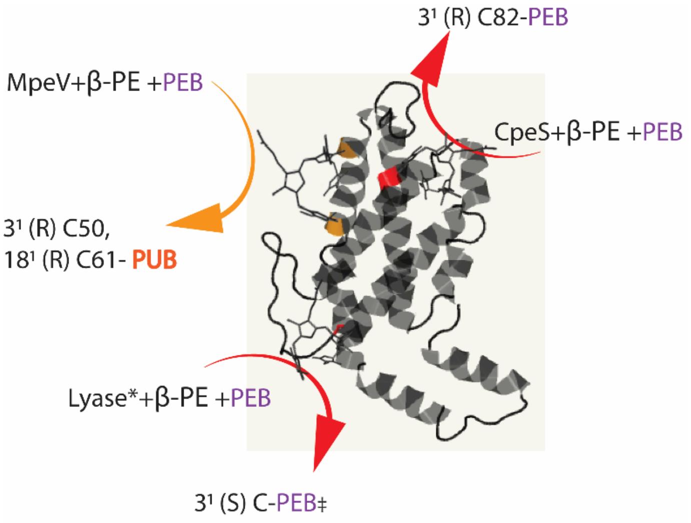

Protein	Cysteine(C)	Bilin (lyase)
β-PEI & β-PEII	82	PEB (CpeS)
β-PEI & β-PEII	50, 61	PUB (MpeV)
β-PEI (β-PEII)	165 (159)	PEB (NC*)

Figure S5. Bilin structure and ligation of RS9916 CpeB. Ribbon diagram generated using Phyre[2] (4), depicting β-subunit of PEI (β-PEI) chromophorylation pattern and lyases involved (bilins are depicted as stick figures). PEB ligated residues highlighted in red and PUB ligated residues highlighted in orange *Lyase not yet characterized (NC), but the candidate is CpeT (7). ‡Bilin configuration is based on characterized homologs (8). The β-subunit of PEII (β-PEII) exhibits a similar chromophorylation pattern with a PEB at C159 (cognate to β-PEI C165).

References

1. Shukla, A., Biswas, A., Blot, N., Partensky, F., Karty, J. A., Hammad, L. A., Garczarek, L., Gutu, A., Schluchter, W. M., and Kehoe, D. M. (2012) Phycoerythrin-specific biln lyase-isomerase controls blue-green chromatic acclimation in marine *Synechococcus*. *Proc. Natl. Acad. Sci. U. S. A.* **109**, 20136-20141
2. Dammeyer, T., Homann, E., and Frankenberg-Dinkel, N. (2008) Phycoerythrobilin Synthase (PebS) of a Marine Virus: Crystal Structures of the Biliverdin Complex and the substrate-free form. *J Biol Chem* **283**, 27547-27554
3. Kronfel, C. M., Hernandez, C. V., Frick, J. P., Hernandez, L. S., Gutu, A., Karty, J. A., Boutaghou, M. N., Kehoe, D. M., Cole, R. B., and Schluchter, W. M. (2019) CpeF is the bilin lyase that ligates the doubly linked phycoerythrobilin on b-phycoerythrin in the cyanobacterium *Fremyella diplosiphon*. *J. Biol. Chem.* **294**, 3987-3999
4. Kelley, L. A., Mezulis, S., Yates, C. M., Wass, M. N., and Sternberg, M. J. (2015) The Phyre2 web portal for protein modeling prediction and analysis. *Nature Protocols* **10**, 845-858.
5. Six, C., Thomas, J.-C., Garczarek, L., Ostrowski, M., Dufresne, A., Blot, N., Scanlan, D. J., and Partensky, F. (2007) Diversity and evolution of phycobilisomes in marine Synechococcus spp.: a comparative genomics study. *Genome Biol.* **8**, R259
6. Wilbanks, S. M., and Glazer, A. N. (1993) Rod structure of a phycoerythrin II-containing phycobilisome I: organization and sequence of the gene cluster encoding the major phycobiliprotein rod components in the genome of marine *Synechococcus* sp. WH8020. *J. Biol. Chem.* **268**, 1226-1235
7. Kronfel, C. M., Biswas, A., Frick, J. P., Gutu, A., Blensdorf, T., Karty, J. A., Kehoe, D. M., and Schluchter, W. M. (2019) The roles of the chaperone-like protein CpeZ and the phycoerythrobilin lyase CpeY in phycoerythrin biogenesis. *Biochim. Biophys. Acta-Bioenerg.* **1860**, 249-561
8. Shen, G., Saunee, N. A., Williams, S. R., Gallo, E. F., Schluchter, W. M., and Bryant, D. A. (2006) Identification and characterization of a new class of bilin lyase: the *cpcT* gene encodes a bilin lyase responsible for attachment of phycocyanobilin to Cys-153 on the beta subunit of phycocyanin in *Synechococcus* sp. PCC 7002. *J. Biol. Chem.* **281**, 17768-17778
